# The contribution of family physicians to African health systems

**DOI:** 10.4102/phcfm.v14i1.3651

**Published:** 2022-07-28

**Authors:** Robert Mash

**Affiliations:** 1 Division of Family Medicine and Primary Care, Faculty of Medicine and Health Sciences, Stellenbosch University, Cape Town, South Africa

**Keywords:** family physicians, Africa, service delivery, health workforce, quality improvement, models of care, primary health care

## Abstract

**Background:**

Africa is the last region to incorporate family physicians into its health systems. They are still a relatively new concept in many countries, small in numbers and deployed in a variety of ways. There is a need for more evidence on their contribution to African health systems to guide policymakers.

**Aim:**

The aim of this study was to review the special collection of short reports on the contribution of family physicians to African health systems, published in the *African Primary Health Care and Family Medicine Journal* in 2021.

**Method:**

Seventeen short reports from eight countries were qualitatively and thematically analysed in ATLAS.ti. Codes, which were derived inductively, were organised into categories according to the World Health Organization’s primary health care monitoring framework.

**Results:**

In the domain of health system determinants, family physicians made little contribution to governance, adjustment to population health needs or financing. They did, however, contribute substantially to the capacity of the health workforce, supply of equipment, functioning of the health information system and use of digital technologies. In the domain of service delivery, they strengthened the model of care and championed systems for improving the quality of care. This translated into improved availability and utilisation of services, core functions of primary care, quality of care and patient safety.

**Conclusion:**

Family physicians described their important contribution to service delivery in district hospitals and primary health care. This should lead to improvements in outcomes and impact for the health system. Their contribution to the concept of resilient facilities and health services needs further exploration.

## Introduction

Africa is probably the last region in the world to incorporate family physicians into its health systems.^1^ Family physicians are medical practitioners with postgraduate training in family medicine, who are usually trained as medical generalists to work in primary health care (PHC). Given the huge need for strengthening district health services in the African region, it seems surprising that health systems are slow to incorporate family physicians.

The supply of family physicians in African countries is typically limited, and many countries have only recently initiated training programmes.^2^ The impact of family physicians is therefore low, and deployment has not yet gone to scale. Policy and decision-makers may be confused about the roles of family physicians in the African context and unsure as to whether the return on investment is worthwhile.^3^ Some assume that the proposed model of care is that of Europe or North America, where family physicians are the main primary care providers, and they dismiss family medicine as impractical and unaffordable. Others would rather invest in the training of medical specialists for central and tertiary hospitals, leaving PHC in the hands of community health workers, nurses and midlevel health workers. Others may even assume that family physicians are most appropriate to the private sector.

The World Organisation of Family Doctors in Africa produced a consensus statement on African family medicine several years ago.^4^ Family physicians, in our context, work in the district or primary hospital as much as in PHC.^5^ They are not usually the first-contact primary care provider but key members of multidisciplinary PHC teams. They may act as clinicians for more complicated patients, consultants to the team, capacity builders and leaders of clinical governance. In the district hospital, they have similar roles and often fill important skill gaps, particularly in rural and remote locations. Whilst African family physicians may share similar values to their colleagues in high-income countries, their roles and required competencies may differ considerably.^6^

It is clear that evidence-based policymaking requires evidence, and there is a need to provide evidence of the contribution of family physicians to African health systems. In South Africa, early research has shown the impact they can make through their various roles, and district managers have recognised their contributions, particularly in district hospitals.^7^ In the region, it is difficult to evaluate their contributions because of the small numbers, unclear responsibilities and varied positioning in the health system. Their strengths as generalists and capacity builders have been identified but mostly in terms of perceptions and qualitative studies.^5^

The *African Primary Health Care and Family Medicine* journal publishes such evidence in the region. The editorial team were aware of the fact that family physicians in different countries were making significant contributions to their health systems but not reporting on these contributions through research projects. Therefore, the journal put out a call for short reports so that family physicians could narrate their stories and make their contributions visible. Where possible, these stories were supported by locally collected evidence. The journal published 17 short reports in 2021 from eight countries (Table 1). This article reviews these reports and the key lessons learnt from this special collection on the contribution of family physicians to African health systems.

**TABLE 1:** Summary of included articles.

First author	Country	Description
Darko^8^	Accra, Ghana	Responding to COVID-19 by providing clinical care and using e-health approaches.
Engmann^9^	Greater Accra, Ghana	How a family physician created a chronic care clinic in a mission hospital to address the increase in noncommunicable diseases.
Hendriks^10^	Eastern Cape, South Africa	The contribution of family physicians to surgical capacity at district hospitals in South Africa.
Ilori^11^	Ibadan, Nigeria	How family physicians created a comprehensive and holistic primary health care service at a health post situated in a home for people with disabilities.
Jenkins^12^	Garden Route, South Africa	A family physician-led initiative to improve values-driven leadership through outreach and support.
Kioko^13^	Kilifi, Kenya	How family physicians led a rural community-orientated primary care project to address HIV stigma.
Kruger^14^	Tshwane, South Africa	The contribution of family physicians to residential mental health care during the COVID-19 pandemic.
Madela-Mntla^15^	Tshwane, South Africa	The role played by family physicians in providing health services for the sheltered homeless during the COVID-19 lockdown.
Mahfud^16^	Somaliland	Strengthening the Somaliland health system by integrating public and private sector family medicine.
Maiga^17^	Mali	The contribution of family medicine to community-orientated health services.
Mailosi^18^	Nkhoma, Malawi	How family physicians based at a mission hospital improved the continuity of primary care and capacity of the primary health care team, particularly for hypertension and diabetes.
Nyamu^19^	Kilifi, Kenya	How family physicians addressed antimicrobial stewardship in relation to upper respiratory tract infections in a rural community-orientated primary care project.
Okori^20^	Northern Uganda	How a family physician contributed to service delivery at a district hospital in a rural and remote location.
Omar^21^	Cape Town, South Africa	The contribution of family physicians to patient safety in people living with HIV and newly started on dolutegravir.
Rabiile^22^	Somaliland	The contribution of family medicine to the health system in Somaliland.
Rossouw^23^	Cape Town, South Africa	The contribution of family physicians in coordinating care and improving access at a small urban district hospital.
Von Pressentin^24^	Mossel Bay, South Africa	The family physician as a primary care consultant – the Mossel Bay experience.

COVID-19, coronavirus disease 2019.

## Methods

This was a review of the special collection on the contribution of family physicians to African health systems in the *African Primary Health Care and Family Medicine* journal. The review is based on a qualitative thematic analysis of published articles. Table 1 summarises the articles included in the review.

All 17 articles were uploaded to ATLAS.ti and codes created inductively as the articles were read. Codes were then organised deductively using the World Health Organization’s framework for monitoring PHC progress and performance.^25^ Codes were organised into code groups based on the structure of the framework, and a report was created for each group. Each report was then interpreted and the findings written immediately, with quotations from the articles to support the interpretation. The findings are presented using the structure of the WHO framework.

The WHO framework is intended to help policymakers to measure the strength of PHC. This article intended to summarise the evidence presented in the special collection for policymakers. Therefore, the framework was seen as an appropriate health systems lens through which policymakers could engage with this evidence.

**FIGURE 1:**
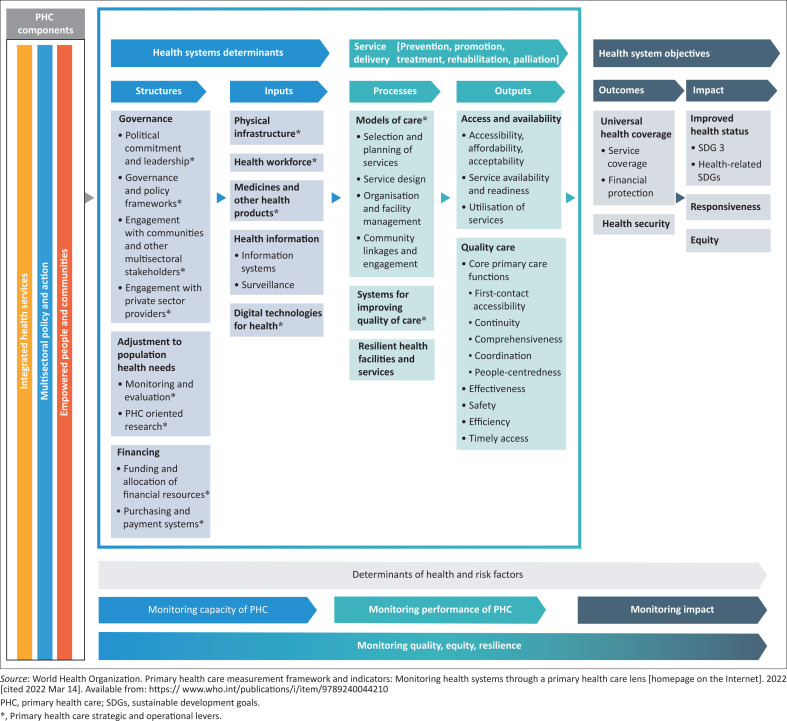
World Health Organization’s framework for primary health care.

The framework is structured in a logic model that flows from structures, inputs, processes, outputs and outcomes to impacts (Figure 1^25^). The structures and inputs are viewed as the necessary health system determinants that are required for the processes and outputs of service delivery. Service delivery is responsible for the achievement of the outcomes and impact under health system objectives. Each domain of the logic model is then broken down into subdomains as shown in Figure 1. Underlying the logic model are the determinants of health and risk factors for that population. The framework’s focus is on measuring the health system determinants and service delivery, and this is also where the family physicians’ reports had their focus.

The author performed the analysis and is also the editor-in-chief of the journal. He was responsible for reviewing and making decisions on all the articles accepted for the special collection. Therefore, he was already familiar with the articles prior to the analysis. Although he conducted the review alone, the analysis was validated by the corresponding authors of the included articles.

## Findings

### Communities and health services contexts

Communities were characterised by high morbidity and mortality with low life expectancy:

The health of the Somaliland population is poor. Life expectancy is estimated at 53 and 56 years for males and females, respectively. One in seven children dies before their fifth birthday, and one in 18 women has a lifetime risk of death during pregnancy^22^. (Somaliland)

Several reports observed an increase in noncommunicable diseases such as diabetes and hypertension. Vulnerable groups, such as older persons, homeless people or those with disabilities, were also present in many communities. Conflict and civil war in several countries had undermined health services. The topography impeded access to PHC in remote and rural areas, where health care workers were also harder to attract and retain. Several of the reports outlined responses to the coronavirus deasese 2019 (COVID-19) pandemic. Many health services were characterised by high workloads, limited time and constrained human resources.

Health workforces were limited in their ability to manage the burden of disease. For example, primary care providers might struggle with managing chronic or complex health conditions, rational prescribing and person-centred care. As a result, patients might bypass PHC or be inappropriately referred:

Challenges in gatekeeping, as well as limitations in resources, time and training, often limit the ability of rural health care providers to appropriately address chronic diseases and complicated cases.^18^ (Malawi)

Many patients [*were*] travelling to Kampala, the capital city, in search of health care services because of the lack of skilled medical personnel.^20^ (Northern Uganda)

According to a report from the Ministry of Health, 70% of cases referred for tertiary care could have been managed at primary care or district hospitals.^18^ (Malawi)

Referral pathways were often difficult because of long distances and waiting times for appointments. Referral centres did not always understand the constraints and context of PHC:

On average, patients waited for 9 months to be informed of their appointment date and 12 months for the actual appointment.^23^ (Cape Town, South Africa)

Infrastructure was often poor with inadequate equipment, medication and supplies:

The only hypertension medications available included hydrochlorothiazide and propranolol; most first-line medications were not available because the health centre did not have a clinical officer or access to appropriate laboratory testing.^18^ (Malawi)

### Influence of family physicians on health system determinants

#### Structures

There was little mention of the structural determinants such as governance, financing and adjustment to population health needs. One exception was in Somaliland, where family physicians pioneered a model of public-private partnership to fill gaps in the health system at the primary hospital level:

However, there are now 10 private primary hospitals in Borama district. The government could improve the delivery of health services in Borama district by contracting private primary hospitals to fill the gap between health centres and the regional hospital.^16^ (Somaliland)

#### Inputs

**Health workforce:** A key area of influence was on the capability of the health workforce in both district hospitals and PHC. Interventions included the running of short courses, introduction of continuing professional development programmes, interactive workshops, training sessions, support and supervision during clinical practice and demonstration of skills, as well as patient presentations and discussions. Family physicians engaged a wide range of health care workers, including community health workers, nurses, midwives, registrars, medical officers and laboratory technicians. In addition, a wide range of clinical conditions and competencies were targeted, such as noncommunicable diseases, childhood illnesses, respiratory tract infections, antibiotic prescribing, HIV stigma, COVID-19, surgery and anaesthesia:

Capacity building of the hospital staff and outreach teams through regular continuous professional development sessions and targeted short course trainings.^20^ (Uganda)

The feedback from staff was used to create continuing professional development on diabetes, hypertension and asthma, which was presented during site visits.^18^ (Malawi)

The purpose of the intervention was to increase adherence to Integrated Management of Childhood Illnesses (IMCI) guidelines for the children under 5 years and adherence to the Kenyan Government Clinical Guidelines for the over-5-year age group.^19^ (Kenya)

Visiting PHC clinics to provide and build capacity on proper use of personal protective equipment, as well as proper procedures for screening and testing for COVID-19.^15^ (Tshwane, South Africa)

Family physicians not only increased the capability of their colleagues, but also boosted motivation and confidence, whilst creating a supportive learning environment:

There are many ways to boost confidence, none of them as effective as having a more experienced doctor next to you or at least on the premises. This is where the availability of a FP in settings where there is no surgeon makes a big difference.^10^ (Eastern Cape, South Africa)

Through the VDL [*values driven leadership*] programme, FM is playing an active role to help create a culture of learning and refection in the health system.^12^ (Garden Route, South Africa)

Family physicians multiply the impact of their own training by teaching others. The family medicine curriculum anticipates these opportunities and includes instruction on how to teach.^22^ (Somaliland)

Family physicians not only improved the capacity of the existing workforce but also helped to prepare future health professionals. In particular, they enabled registrars to be part of the health services and to engage with service-learning innovations. For example, registrars created a new service for people with disabilities, implemented community-orientated primary care projects and built the capacity of PHC teams to manage noncommunicable diseases. Family medicine training programmes extended into rural areas and helped to improve the capacity of rural health services:

This provides [*the registrars*] with a vital opportunity to practice integrating primary care practice and public health. They also develop their leadership skills by developing an intervention for a locally identified health problem.^13^ (Kenya)

[*This*] demonstrates how primary care facilities can improve NCD management, the FM registrars … are distinguishing themselves as capable and efficient leaders of health care.^18^ (Malawi)

Family physicians also contributed to the training of interns and medical students. Such training could cultivate an interest in social responsibility and service to remote and vulnerable populations:

The influence of the new Family and Community Medicine programme also demonstrates that early exposure of health science students to primary health care and the concept of social responsibility is possible and productive, as it promotes learner involvement with vulnerable and/or remote populations.^17^ (Mali)

**Improved supply of medicine and other products:** Family physicians advocated for the equipment needed to improve the comprehensiveness of services and quality of care. The type of equipment varied depending on the setting but included glucometers, oxygen concentrators and oximeters, surgical equipment, anaesthetic machines and ultrasound.

**Health information:** Family physicians found the importance of health information and made numerous innovations to improve these systems. They helped with the design of new tools to collect data, standardised the data collected, supervised the quality of data collected and performed data analysis and reporting. Support for informational continuity in the clinical setting ranged from the acquisition of an electronic medical record system in the private sector to introduction of simple paper-based registers in the public sector:

Following the acquisition of an electronic health record system in September 2020, patient data has become readily available for the clinical team to analyse and support informational continuity during ongoing care.^9^ (Ghana)

This information was shared with the team, who decided to record and track individuals who presented with elevated BP or glucose levels in a register which was provided by the FM team, which also included appropriate treatment protocols.^18^ (Malawi)

**Digital technologies for health:** Family physicians reported a variety of ways in which they had innovated or harnessed technology to assist with health information and service delivery. This commitment to using technology was amplified during the COVID-19 pandemic. However, the use of technology was limited by disruptions in electricity supply, slow Internet connections and poor digital literacy:

FPs to go beyond their normal clinical roles by immersing themselves into additional activities, including data management, research and effective utilisation of technology.^15^ (Tshwane, South Africa)

Technology was used to assist with coordination of care between primary and secondary levels. For example, a family physician was the gatekeeper for a new referral system to cardiology using e-mail; in another setting, the Vula programme [*it is an app and not a programme*] was used for referrals to specialist care and the Mpilo programme [*it is an app and not a programme*] to coordinate transport for the homeless:

The FPs presented the data to the head of the cardiology service and proposed a new e-mail referral system. The FPs would screen all referrals, which could significantly reduce the number of referrals.^23^ (Cape Town, South Africa)

Family physicians also championed the use of technology to improve health information. One of the family physicians introduced an electronic system to collect information on surgical cases at a district hospital. This helped to monitor patient safety and motivate for equipment:

With elective surgical lists and a new digitalised monitoring system in place, we could track how many cases we were doing, who was doing them and plan better for future procedures.^10^ (Eastern Cape, South Africa)

Finally, family physicians harnessed the technology to improve communication and consultation with patients. In Ghana and South Africa, family physicians used a variety of digital solutions such as m-health, a telephonic hotline for queries, virtual education on COVID-19 via Facebook Live and videoconferencing for consultations. Family physicians were also able to consult and support other health care workers using WhatsApp and Zoom videoconferencing for virtual ward rounds. Technology could improve the efficiency of consultant-level support from a distance:

In June 2020, NMC [*Nyaho Medical Centr e*] and Clearspace Labs developed a telemedicine platform. “Virtual Care” used an encrypted video consultation which greatly enhanced care for people with COVID-19 infection as compared to the previously used telephone consultation.^8^ (Ghana)

Zoom provided the opportunity to do daily “virtual ward rounds” where patients could be discussed and advice given … on the use of blood tests, oxygen concentrators, antibiotics and steroids.^14^ (Tshwane, South Africa)

### Contribution of family physicians to service delivery

#### Processes

**Models of care:** Family physicians influenced the selection, organisation and delivery of services in PHC and district hospitals. In one setting, they reinforced the PHC approach with an emphasis on health promotion and disease prevention, whilst in another they extended services to vulnerable groups, such as the homeless and people with mental health disorders. They were often involved in the design of new services, particularly for chronic conditions. Family physicians championed family-orientated primary care and a willingness to conduct home visits. In addition, they helped to improve cohesion and coordination of care between PHC, district hospitals and regional or tertiary hospitals:

The provision of family-centred care has fostered the implementation of domiciliary services (such as home visits) and family-level interventions (such as family conferencing for crisis resolution).^11^ (Nigeria)

The implementation of the chronic care clinic was the initiative of the author. This was after a family practice needs assessment in the facility and also the growing need for specialised care for patients with chronic disease.^9^ (Ghana)

Subsequent to the meeting, both FPs and medical officers reported an improved referral experience to VHW [*Victoria Hospital in Wynberg*]. Improving coordination of care across the primary–secondary care interface requires building relationships of mutual respect and trust and overcoming ‘professional tribalism.^23^ (Cape Town, South Africa)

Family physicians made an important contribution to services for health promotion and disease prevention, particularly in relation to noncommunicable chronic diseases, cancers, reproductive and sexual health, substance abuse and COVID-19. Family physicians also improved services for emergency, maternal and neonatal care, as well as for survivors of sexual assault. In at least one setting, palliative care was a key initiative:

The FM Department has been instrumental in the development of the antibiotic stewardship programme, the palliative care service, the emergency centre service, the institutional training programme, the women’s health and Thuthuzela (Sexual Assault) service, the response to tuberculosis, occupational health, wellness and a smoking cessation clinic for staff.^12^ (Garden Route, South Africa)

The fact that 15 [*family medicine*] graduates are routinely doing C-sections contributes to achieving the highest priority objective of the health services: to reduce maternal, neonatal and child mortalities.^22^ (Somaliland)

Family medicine principles resonated with a more collaborative and supportive leadership and management style. Typically, they worked alongside managers; however, in a few places they were appointed as medical or clinical managers. Mostly family physicians were involved in leading their clinical teams and in leading projects to improve the model of care. Leadership was often mentioned in the context of working with teams and improving their function:

Family physicians and health managers who live according to the principles of FM have enabled traditional hierarchical and mechanistic organisational culture to slowly transform.^12^ (Garden Route, South Africa)

These changes required the collaboration and engagement of all clinical, support and managerial staff, but the FP was a critical catalyst in making these changes.^20^ (Northern Uganda)

There was a particular emphasis on supporting the implementation of community-orientated primary care (COPC). In terms of COPC, family physicians reported leading proactive community outreach initiatives. In addition, they championed the identification of and response to local health needs, in collaboration with the health care teams and community. They also role modelled an approach that focused on the population at risk within a delineated geographical area, rather than just the patients attending the health facility. There were several examples of working with and supporting community health workers:

Community entry and engagement were harnessed through community outreach programmes organised by the team led by family medicine residents and consultants. Such outreaches targeted at traders, artisans, older persons and school children have benefitted many people with health promotion information about good health-seeking behaviour, chronic disease prevention and care, human immunodeficiency virus (HIV) infections, education on risky health behaviours and cancer screening.^11^ (Nigeria)

This report presents an example of how the community can be involved in designing an intervention for a locally identified health problem, through the process of COPC under the guidance of family physicians, whilst at the same time providing a valuable learning experience for medicine residents.^13^ (Kenya)

There were a few examples of family physicians working in regional hospitals. As medical generalists, they were used to plug gaps in the services when other specialists were absent. Whilst they could make a useful contribution at this level, it was not the ideal setting for them to contribute. Almost all family physicians were deployed in the district health services, and it was argued that increasing their numbers would have an even greater impact:

Because of the specialist compartmentalisation of the regional hospital, the four family physicians were not able to practise comprehensive family medicine. … Ultimately, however, as other specialists become available for the regional hospital, the health system will function better with family physicians working at the primary hospital.^16^ (Somaliland)

The phenomenon of a sole FP per subdistrict is not sustainable and limits the FP’s ability to strengthen the PHC service outside the district hospital, as the single FP needs to divide his or her contribution between the two.^24^ (Mossel Bay, South Africa)

**Systems for improving the quality of care:** Family physicians brought a systems perspective of the health services. Rather than accepting ‘business as usual’, they proactively looked for ways to improve. Examples included a focus on improving access to care, coordination of care and surgical capacity:

These three interventions demonstrate clearly how FPs may use their understanding and clinical experience of the local burden of disease, system inequities, as well as available data to advocate for improved patient care access and coordination.^23^ (Cape Town, South Africa)

Many of the reports illustrated how family physicians used their research skills to evaluate and improve service delivery. These included evaluating antibiotic prescribing, outreach to primary care, referrals to secondary care, introduction of new antiretroviral medication, understanding the needs of homeless people and the impact of leadership training. These small-scale evaluations were not formal research projects but demonstrated how family physicians applied their skills in formulating relevant questions, collecting and analysing data, interpreting the results with their teams and acting on them:

This evaluation revealed excessive over-prescription of antimicrobials in the management of URTI in both the under-5-year age group and the over-5-year age group (including adults), with 70% – 90% of the patients receiving antibiotics.^19^ (Kenya)

In 2017, FPs in the district hospital reviewed the annual mortality data between 2015 and 2017 and found that more than a third of deaths (39%) were cardiac in origin. A cross-sectional audit was conducted in the outpatient department during September 2017, which highlighted a significant problem for cardiac patients in accessing definitive care.^23^ (Cape Town, South Africa)

Family physicians were instrumental in leading clinical governance activities and improving the quality of care. Activities included introduction of new services, teaching and training, development of local clinical protocols, implementation of clinical guidelines, audit and feedback, introduction of morbidity and mortality meetings and facilitating reflection on health information. In one setting, family physicians in a regional hospital performed outreach to local district hospitals and primary care facilities, as well as organising in-reach of staff to the regional hospital for upskilling. One of the family physicians focused on a more integrated approach to clinical governance across levels of care. There was also a commitment to continuous quality improvement and a more systematic approach:

The family medicine faculty members and registrars brought to the hospital evidence-based standards of care, professionalism, morning report, morbidity and mortality review, improved documentation, daily rounds, regular teaching and dependable backup at night and on weekends.^16^ (Somaliland)

Quality assessment evaluation (meaning the traditional quality evaluation of services) has been replaced with continuous quality improvement, which is a more dynamic approach. This includes community-orientated teaching methods and continuous improvement of services through community collaboration and applied research projects.^17^ (Mali)

**Resilient health facilities and services:** The recent COVID-19 pandemic is a good example of a significant challenge to the resilience of district health services. The reports demonstrated that family physicians made an important contribution to helping services respond and adapt. For example, in South Africa, family physicians helped to maintain services, contain COVID-19 and respond to the needs of the homeless and those in residential care:

The relevance of this report is that it examines the roles that can be played by FPs during public health crises and highlights the importance of their role within community-based multidisciplinary teams.^15^ (Tshwane, South Africa)

#### Outputs

**Access and availability:** A key impact of family physicians was through improved availability and utilisation of services. Family physicians, with their wide skill set, enabled a more comprehensive range of services at the local level. They carried this out through direct clinical care at the district hospital, outreach to the PHC clinics and even via telehealth during the COVID-19 pandemic:

Importantly, this family physician outreach service and consultant clinics at primary health care facilities improved the patient care experience by improving access to specialised care, coordinated by the FP, closer to their home and community.^24^ (Mossel Bay, South Africa)

The family physician-led innovations described here illustrate that it is possible to enhance access to equitable, efficiently coordinated and quality patient-centred care closer to their patients’ homes.^23^ (Cape Town, South Africa)

Family physicians were also instrumental in improving access to medication. In Malawi, family physicians successfully advocated for the supply of medication to primary care facilities to treat noncommunicable diseases. In South Africa, family physicians were key to organising the home delivery of medication during COVID-19, whilst in rural Uganda, this involved establishing community distribution points for anti-retroviral drugs:

Innovative HIV care and treatment by setting up 33 community distribution points for anti-retroviral (ARV) drugs. Each point serves an average of 70 patients monthly, which greatly improved the access to ARVs for people living with HIV from 2123 to 5200 over a 5-year period.^20^ (Northern Uganda)

Minor surgical procedures were made available in PHC, whilst more major surgery was possible at the district hospital through the presence of family physicians and registrars. Utilisation of maternal care improved with the presence of a family physician. Family physicians were also successful in improving access to imaging and laboratory investigations at the local level:

As a result of these improvements [*led by the family physician*], in-hospital neonatal mortality decreased from 53% to 10%, major surgeries performed increased from 530 to 1620, maternity admissions increased from 2700 to 3313 and babies delivered increased from 1872 to 2967.^20^ (Northern Uganda)

During my last year at the [*rural district*] hospital, we completed 966 cases in the one available theatre. All had theatre notes, surgical safety checklists and information on all these procedures were available digitally.^10^ (Eastern Cape, South Africa)

Equipping and expanding the laboratory to improve the services. An application for accreditation was then made and it is now the only internationally accredited laboratory in the region.^20^ (Northern Uganda)

Family physicians led numerous initiatives to improve access to care for marginalised or neglected groups in their communities such as people with disabilities, older persons, homeless people and those with intellectual impairment and mental health disorders. Family physicians also led initiatives to improve rural health care:

One of the key interventions involving vulnerable persons attracted to the integrated services provided at the centre is the Older Person Wellness Program (also known as the senior citizens’ club).^11^ (Nigeria)

This report describes the virtual support provided by two family physicians to a mental health care institution in Tshwane, during the first wave of the pandemic in 2020. [*This*] is a residential care facility that offers medical and residential care to 160 severe and profoundly disabled adults.^14^ (Tshwane, South Africa)

In addition, the presence of a family physician could improve access to the next level of expertise by ensuring appropriate referrals and improving the timeliness of appointments. Their specialist training enabled them to negotiate and collaborate with specialists at the referral centre. In one example, family physicians were able to directly book patients for CT scans at the referral hospital, because they were recognised as having appropriate clinical decision-making skills.

**Quality care:** The previous examples of clinical governance activities illustrated tangible improvements in access to care, comprehensiveness, coordination, continuity, person-centredness, equity, adherence to clinical standards, responsiveness to patients’ needs, safety and timeliness of access to secondary care.

## Discussion

### Summary of key findings

The contribution of family physicians was mainly in the domain of service delivery in both district hospitals and PHC. They made a substantial contribution to the models of care and to establishing systems of quality improvement and patient safety. This translated into improved access to and availability of services close to communities. They also improved all of the core primary care functions such as first-contact accessibility, comprehensiveness, coordination, continuity and person-centredness. There were several examples of improved effectiveness, efficiency, equity, safety and timely access to the next level of expertise. Their contribution to resilient health services and facilities was not as evident.

They had some influence on the health system determinants, particularly the inputs to service delivery. In this domain, their main contribution was in improving the capability and motivation of the workforce and creating a supportive learning environment. In addition, they supported the development of health information systems and the use of digital technologies. They had little engagement with the more structural components of the health system.

### Discussion of key findings

These reports from multiple countries in East, West and Southern Africa illustrate that family physicians improve service delivery through three key roles:

- as clinicians and consultants to health care teams
- as capacity builders of health care teams and clinical trainers to future health professionals
- as leaders of clinical governance who improve the quality of care and patient safety.

These roles were conceptualised previously,^26^ but are clearly illustrated and confirmed in these reports from diverse contexts.

Family physicians appear to bring a systems viewpoint, leadership skills and a sense of agency to strengthening district health services. These higher-order competencies are vital to health care teams at district hospitals and in PHC. Whilst it is clear that family physicians make their contribution through teamwork, their leadership may well be critical to improving service delivery. This review of short reports provides additional evidence of the need for including family physicians in health care teams. In many cases, the family physician was located at the district hospital but reaching out to support the PHC services in the catchment area.

The WHO framework document notes that family physicians are of particular relevance to PHC,^25^ and the World Health Assembly has previously included them in its definition of the PHC team.^27^ This review provides concrete examples of their contribution in the African context in both low- and middle-income countries. Most governments in the African region have signed the Astana Declaration and made a renewed commitment to strengthen PHC.^28^ This review shows the promise of family physicians as an intervention across the whole service delivery domain.

It is not surprising that the reports showed little impact on the structural domain of the health system, as this is usually the responsibility of national and subnational policy- and decision-makers. The inputs required for service delivery are also usually the responsibility of managers at district and facility level.

The one subdomain of service delivery where their contribution was less was that of resilient services and facilities. The emphasis on resilience is relatively new and is particularly relevant in the face of challenges such as the COVID-19 pandemic. In the future, climate change and extreme weather events will also test the resilience of health workers in PHC.^29^ Family physicians may need to develop new alliances with the disciplines of public health and emergency medicine to prepare for and adapt to the likelihood of these events.

The reports commented only briefly on the outcomes and impacts of the changes to service delivery. This is not surprising as changes to these would be longer term and require formal research to measure. Family physicians were able to report on what they had done, seen and, to some extent, measured at the local level. The purpose of the data collection was to allow family physicians to narrate their stories without the need for a formal research study.

### Limitations

The review is based on 17 short reports that intended to showcase the contribution of family physicians. These reports may not be typical of all family physicians in the region and could be overly optimistic. Nevertheless, the reports came from a wide variety of countries, both urban and rural contexts, as well as from low- and middle-income settings. The reports did not have the scientific validity of a well-formulated research study, although family physicians did provide supportive evidence when available and described their contributions in detail.

### Implications and recommendations

By synthesising the key themes from these multiple short reports, this review provides a more reliable and valid analysis of the contributions of family physicians than one report by itself. The picture that emerges suggests that family physicians can make a substantial difference to the performance of PHC and district health services. The evidence distilled here should encourage policymakers to invest in the training and deployment of family physicians in their health systems. Universal health coverage requires both coverage and quality.^30^ Whilst health care workers at other levels, such as community health workers, can provide some of the needed coverage, family physicians are needed to ensure quality and the availability of comprehensive services at the community level.

## Conclusion

Family physicians can make a substantial contribution to African PHC and district-level service delivery. They improve the models of care, enhance systems for quality improvement, improve access and availability of care and build the capacity of the workforce. They particularly strengthen the core primary care functions of first-contact accessibility, comprehensiveness, coordination, continuity and person-centredness. This review adds additional evidence that in the African context, family physicians are an essential human resource in health care teams. Policymakers on the continent should consider whether family physicians can help them to realise their political commitments to strengthening PHC.
